# The Molecular Basis and Biologic Significance of the β-Dystroglycan-Emerin Interaction

**DOI:** 10.3390/ijms21175944

**Published:** 2020-08-19

**Authors:** Wendy Lilián Gómez-Monsivais, Feliciano Monterrubio-Ledezma, Jazmin Huerta-Cantillo, Ricardo Mondragon-Gonzalez, Alma Alamillo-Iniesta, Ian García-Aguirre, Paulina Margarita Azuara-Medina, Raúl Arguello-García, Jhon Erick Rivera-Monroy, James M. Holaska, Jesús Mauricio Ernesto Hernández-Méndez, Efraín Garrido, Jonathan Javier Magaña, Steve J. Winder, Andrea Brancaccio, Ivette Martínez-Vieyra, Fernando Navarro-Garcia, Bulmaro Cisneros

**Affiliations:** 1 Department of Genetics and Molecular Biology, Centro de Investigación y de Estudios Avanzados del Instituto Politécnico Nacional (CINVESTAV), 07360 Mexico City, Mexico; wlgomez@cinvestav.mx (W.L.G.-M.); eduardo.monterrubio@cinvestav.mx (F.M.-L.); rmondragon90@gmail.com (R.M.-G.); aalamilloi@cinvestav.mx (A.A.-I.); ian.garcia@cinvestav.mx (I.G.-A.); pauazmd@gmail.com (P.M.A.-M.); rag@cinvestav.mx (R.A.-G.); mauriciohermen91@hotmail.com (J.M.E.H.-M.); egarrido@cinvestav.mx (E.G.); 2 Cell Biology Department, Centro de Investigación y de Estudios Avanzados del Instituto Politécnico Nacional (CINVESTAV), 07360 Mexico City, Mexico; jazmin.huerta@cinvestav.mx (J.H.-C.); enavarro@cinvestav.mx (F.N.-G.); 3 Department of Molecular Biology, Faculty of Medicine, GZMB, Georg-August University, 37073 Göttingen, Germany; jhonriv@gmail.com; 4 Laboratorio Instrumental de Alta Complejidad, Universidad de La Salle, 111311 Bogotá, Colombia; 5 Department of Biomedical Sciences, Cooper Medical School of Rowan University, 401 S Broadway, Camden, NJ 08028, USA; holaska@rowan.edu; 6 Laboratory of Genomic Medicine, Department of Genetics, National Rehabilitation Institute-Luis Guillermo Ibarra Ibarra (INR-LGII), 14389 Mexico City, Mexico; 7 Departamento de Bioingeniería, Escuela de Ingeniería y Ciencias, Instituto Tecnológico y de Estudios Superiores de Monterrey-Campus Ciudad de México, 14380 Ciudad de México, Mexico; 8 Department of Biomedical Science, University of Sheffield, Western Bank, Sheffield S10 2TN, UK; s.winder@sheffield.ac.uk; 9 School of Biochemistry, University of Bristol, Bristol BS8 1TD, UK; 10 Institute of Chemical Sciences and Technologies “Giulio Natta” (SCITEC), 00168 Roma, Italy; andrea.brancaccio@cnr.it; 11 Laboratory of Hematobiology, Escuela Nacional de Medicina y Homeopatía, Instituto Politécnico Nacional, 07320 Ciudad de México, Mexico; ivette0381@yahoo.com.mx

**Keywords:** β-dystroglycan, emerin, nuclear envelope, Emery-Dreifuss muscular dystrophy, surface plasmon resonance assay, proteasome

## Abstract

β-dystroglycan (β-DG) assembles with lamins A/C and B1 and emerin at the nuclear envelope (NE) to maintain proper nuclear architecture and function. To provide insight into the nuclear function of β-DG, we characterized the interaction between β-DG and emerin at the molecular level. Emerin is a major NE protein that regulates multiple nuclear processes and whose deficiency results in Emery–Dreifuss muscular dystrophy (EDMD). Using truncated variants of β-DG and emerin, via a series of in vitro and in vivo binding experiments and a tailored computational analysis, we determined that the β-DG–emerin interaction is mediated at least in part by their respective transmembrane domains (TM). Using surface plasmon resonance assays we showed that emerin binds to β-DG with high affinity (KD in the nanomolar range). Remarkably, the analysis of cells in which DG was knocked out demonstrated that loss of β-DG resulted in a decreased emerin stability and impairment of emerin-mediated processes. β-DG and emerin are reciprocally required for their optimal targeting within the NE, as shown by immunofluorescence, western blotting and immunoprecipitation assays using emerin variants with mutations in the TM domain and B-lymphocytes of a patient with EDMD. In summary, we demonstrated that β-DG plays a role as an emerin interacting partner modulating its stability and function.

## 1. Introduction

The nuclear envelope (NE) of animal cells is composed of three distinct compartments: a double nuclear membrane (the inner and outer nuclear membranes), the nuclear pore complex (NPC) and the nuclear lamina. The NE acts as a hub for a variety of nuclear processes, including gene expression, DNA repair and chromatin organization [1]. It contains several integral and peripherally associated proteins, which modulate its function and integrity, via interaction with the chromatin and the nuclear lamina [2]. The nuclear lamina is a dense meshwork of lamin filaments, comprising A-type-lamins (A and C) and B-type lamins (B1 and B2) [1,2]. Coupling of NE to cytoskeletal elements is enabled by the Linker of the nucleoskeleton and cytoskeleton (LINC) complex, which modulates force transmission across the nuclear boundary [3]. The LINC complex comprises Sad1/UNC-84 (SUN)-domain containing proteins, which span the inner nuclear membrane and nesprins (nuclear envelope spectrin-repeat proteins), located in the outer nuclear membrane) [3].

Despite the critical importance of the NE, its protein composition is not yet completely characterized, and it is likely that additional protein components remain to be unveiled. In this regard, we recently described that β-dystroglycan (β-DG; the β-subunit of the dystroglycan [DG] complex) is a new NE protein in myoblasts [4]. DG is an integral membrane receptor that links the extracellular matrix with the actin-based cytoskeleton [5], which is composed of α-DG and β-DG subunits derived from a single gene product upon proteolytic cleavage [6,7]. To reach the NE, β-DG undergoes retrograde trafficking from the cell surface to the nucleus, via the membranous endosome-endoplasmic reticulum (ER) route. Exit of β-DG from the ER membranous environment is facilitated by the Sec61 translocon complex [8] and nuclear translocation is completed through recognition of the β-DG nuclear localization signal by importins α2/β1 [9].

Within the nucleus β-DG forms a complex with the NE proteins emerin and lamins A/C and B1, to preserve nuclear structure and function [4,10] and probably to regulate gene expression [11]. It is possible that nuclear trafficking of β-DG connects functionally the plasma membrane with the NE, via an as yet undefined mechanism, thus allowing the cell to orchestrate nuclear activity in response to external stimuli [8]. A better characterization of the interaction between of β-DG and its NE interacting partners will help to decipher the molecular basis underlying its role in critical nuclear processes. In this study, we describe in depth the interaction of β-DG with emerin, a major NE protein whose deficiency causes Emery–Dreifuss muscular dystrophy (EDMD) [12,13] and provide compelling evidence showing the biologic significance of their interplay.

## 2. Results

### 2.1. The Interaction between β-DG and Emerin Requires Their Respective Transmembrane Domains

**Figure 1:**
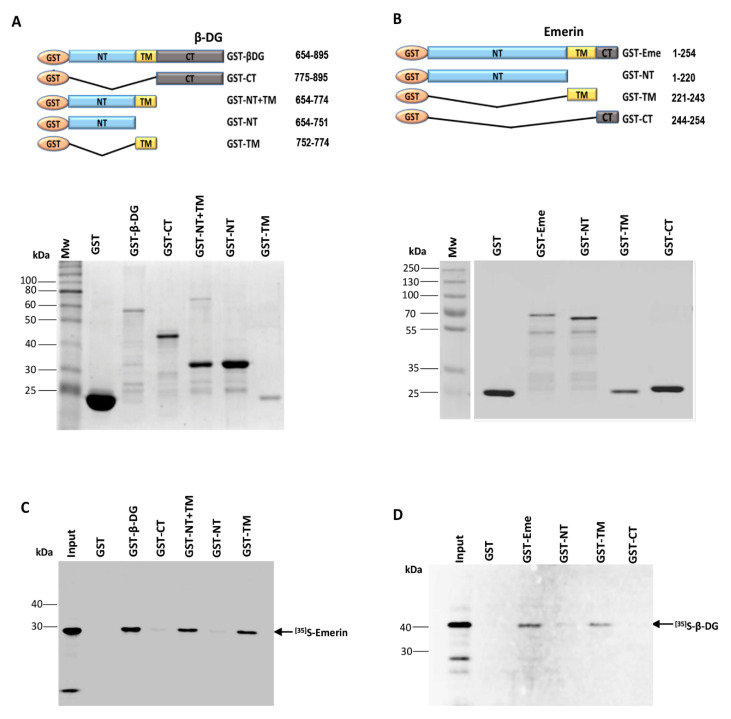
Mapping of protein domains involved in the interaction between β-DG and emerin. (**A**) Top panel. Schematic representation of GST and GST-fusion proteins containing full-length β-DG or its separate domains, N-terminal domain (NT); C-terminal domain (CT), transmembrane domain (TM) and NT+CT domains. The numbers on the right indicate the amino acid residues of β-DG contained in each construct. Bottom panel. GST-tagged β-DG proteins were expressed in *E. coli*, purified using glutathione-Sepharose beads and visualized by SDS-PAGE followed by Coomassie blue staining. Mw, Protein molecular weight markers; (**B**) Top panel. Scheme showing GST and GST-fusion proteins containing full-length emerin or its separate domains, N-terminal (NT); C-terminal domain (CT) and transmembrane (TM) domains. The amino acid residues of emerin present in each construct are indicated on the right. Bottom panel. Representative Coomassie blue-stained gel showing the expression of GST and GST-tagged emerin proteins, expressed in bacteria and further purified as per (**A**). Mw, protein molecular weight markers; (**C**) GST and GST-tagged β-DG proteins immobilized on glutathione-Sepharose beads were incubated with in vitro labeled ^35^S-emerin to perform pull-down assays. Phosphorimaging results documenting interaction of ^35^S-emerin with GST–β-DG, GST–NT-TM and GST–TM, but not with GST–NT, GST-CT or GST alone are shown; (**D**) GST and GST-tagged emerin proteins previously immobilized on glutathione-Sepharose beads were incubated with in vitro translated ^35^S-β-DG to carry out in vitro interaction assays. Interaction of ^35^S-β-DG with GST–emerin and GST–TM, but not with GST–NT, GST-CT or GST alone was revealed by phosphorimaging analysis. (**C**,**D**) The input lanes correspond to 10% of the reticulocyte reaction used in the binding assays.

An association between β-DG and emerin was previously shown in immortalized mouse myoblasts (C2C12 cells) [4]; thus, we set out to identify the specific domain (s) on each protein involved in this interaction. First, the ability of different domains of β-DG to interact with emerin was examined in vitro using GST-based pull-down assays. Full-length β-DG as well as its separate domains, namely N-terminal (NT), transmembrane (TM), C-terminal (CT) and NT plus TM (NT+TM) were expressed and purified in *E. coli* as GST fusion proteins (Figure 1A), while emerin was produced and labeled with [^35^S]-methionine using the pSG5–emerin vector (Table 1) and a coupled transcription–translation rabbit reticulocyte lysate system (Supplementary Figure S1, left panel). Equal amounts of GST fusion proteins bound to glutathione-Sepharose beads, were incubated with [^35^S]-labeled emerin and subjected to pull-down analysis. As evident by SDS-PAGE and autoradiography analyses, interacting complexes were recovered in the precipitated fraction of GST–β-DG, GST–TM and GST–NT+TM, but not in those of GST–NT, GST-CT or GST alone (Figure 1B). These results indicated that the TM of β-DG is necessary and sufficient for binding emerin. To identify which domain of emerin mediates its interaction with β-DG, reciprocal GST pull-down assays were carried out by expressing GST alone, GST–emerin (full-length) or its isolated NT, TM and CT domains in bacteria (Figure 1C, incubating them with ^35^S-labeled β-DG (Supplementary Figure S1, right panel). β-DG was produced and labeled using pSG5–β-DG vector (Table 1) and the transcription–translation rabbit reticulocyte lysate system. Both GST–emerin and its isolated TM domain (GST–TM) had the ability to interact with β-DG, while GST–NT recovered only a trace of labeled β-DG and both GST-CT and GST alone failed to bind β-DG (Figure 1D). Collectively these data demonstrated in vitro interaction between β-DG and emerin, likely to take place through the association of their respective transmembrane domains.

**Figure 2:**
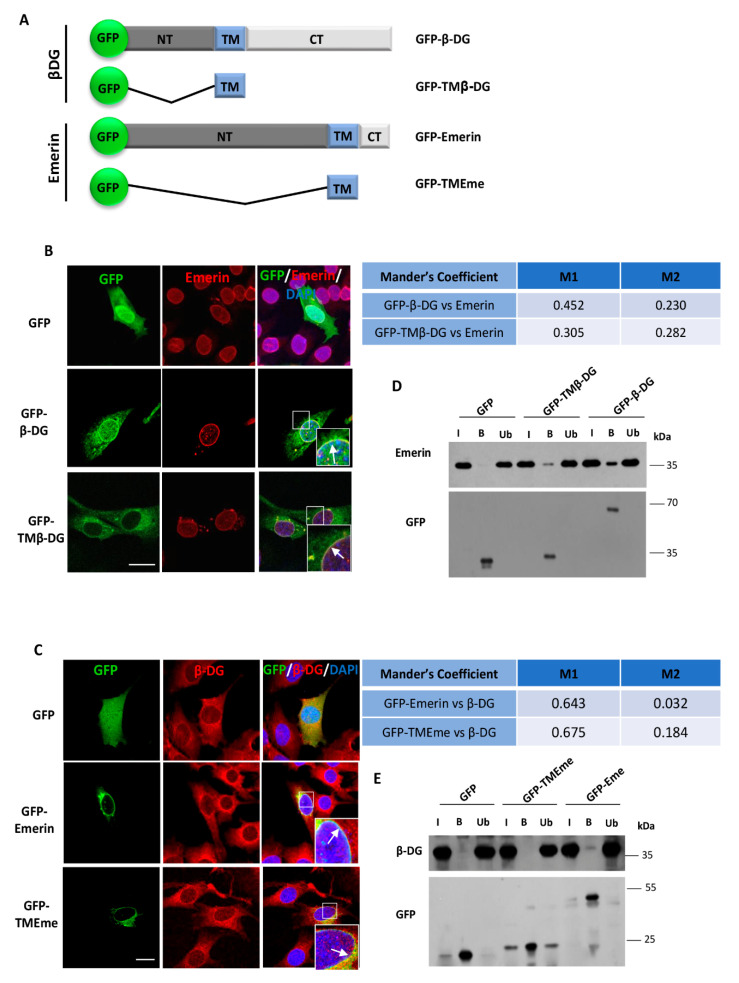
Transmembrane domain of β-DG interacts with emerin in intact cells. (**A**) Schematic representation of GFP-fusion proteins containing full-length β-DG (GFP–β-DG) or its separate transmembrane domain (GFP–TMβ-DG), as well as full-length emerin (GFP–emerin) or its separate transmembrane domain (GFP–TM–Eme). (**B**,**C**) C2C12 cells were transiently transfected to express GFP alone or the above mentioned GFP-tagged proteins. At 24 h post-transfection, the cells were immunostained for β-DG or emerin as indicated and counterstained with DAPI to decorate nuclei, prior to be analyzed by confocal microscopy. Representative single optical Z-sections are shown Scale bar, 10 µm. The Manders overlap coefficient was calculated on double labeling immunofluorescence (vs, versus); (**B**) M1 denotes the signal of GFP–β-DG or GFP–TMβ-DG coincident with endogenous emerin signal over its total intensity, while M2 denotes the signal of endogenous emerin coincident with GFP–β-DG and GFP–TMβ-DG signal over their total intensity (right chart); (**C**) M1 denotes the signal of GFP–emerin or GFP–TM–Eme coincident with endogenous β-DG signal over its total intensity, while M2 denotes the signal of endogenous β-DG coincident with GFP–emerin and GFP–TM–Eme signal over their total intensity (right chart). (**D**,**E**). Lysates from transfected cells were subjected to immunoprecipitation using the GFP-trap system and the precipitated proteins were visualized by SDS-PAGE/WB analysis using anti-emerin, anti-β-DG or anti-GFP antibodies. Input (I) correspond to 5% of lysates prior to immunoprecipitation. B, bound fraction; Ub, unbound fraction.

To demonstrate the implication of the TM domains for β-DG–emerin interaction in a membrane environment in cells, vectors expressing GFP fused to the TM of β-DG (GFP–TMβ-DG) or to the TM of emerin (GFP–TM–Eme) were prepared to carry out localization and GFP-based immunoprecipitation (IP) assays. C2C12 myoblasts were transiently transfected to express GFP alone, GFP–β-DG, GFP–TMβ-DG, GFP–emerin or GFP–TM–Eme (Figure 2A) and then, subcellular distribution of all the recombinant proteins was analyzed by confocal microscopy. GFP–β-DG and GFP–TM–β-DG, as well as GFP–emerin and GFP–TM–Eme were targeted also to the NE to a certain extent, where colocalized with endogenous emerin and β-DG, respectively (Figure 2B,C). These observations were supported by Mander’s overlap coefficient analysis (Figure 2B,C, right charts). GFP alone distributed between the cytoplasm and the nucleus and was not colocalized specifically with endogenous emerin or β-DG. IP assays demonstrated that endogenous emerin was pulled down by GFP–β-DG (positive control) and to a lesser extent by GFP–TMβ-DG (Figure 2D), which implies that the TM domain of β-DG maintains its ability to interact with emerin in intact cells. Conversely, IP assays showed that GFP–emerin (positive control) immunoprecipitated only trace amounts of endogenous β-DG, while GFP–TM–Eme failed to interact with endogenous β-DG (Figure 2E). It is possible that the presence of GFP affects the interaction between GFP–TM–Eme and endogenous β-DG. This latter finding agrees with the observation that GFP–TM–Eme was localized less robustly with endogenous β-DG (Figure 2C, lowest panel) than in the reciprocal experiment (Figure 2B. lowest panel). Alternatively, these findings also may implicate that the TM of emerin is unable by itself to maintain interaction with β-DG in intact cells and that adjacent segments of the protein (NT and/or CT domains) are required.

**Figure 3:**
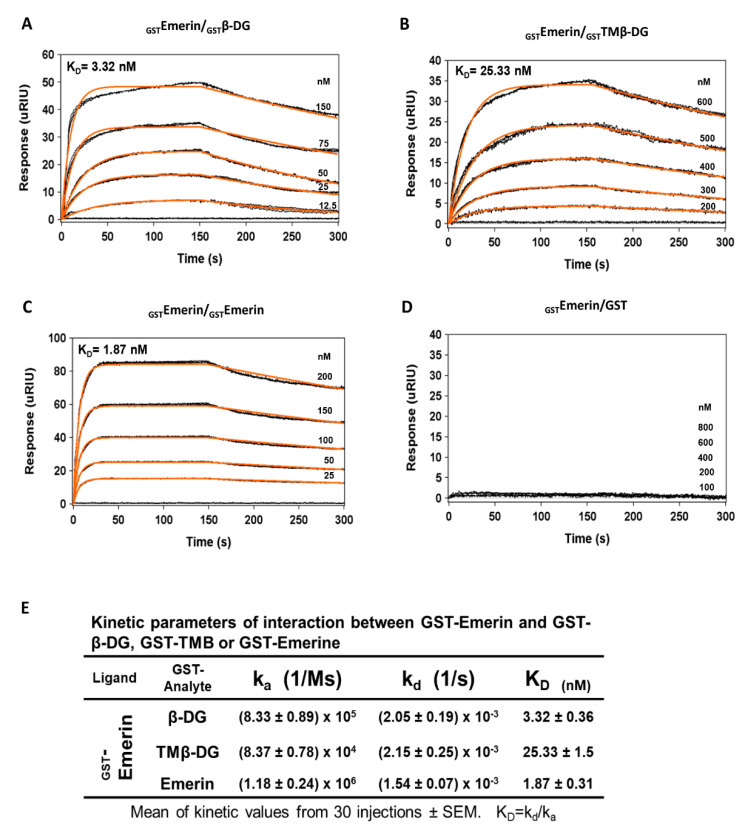
Affinity of emerin for β-DG and the TM domain of β-DG. Surface plasmon (**A**) resonance (SPR) assays were performed to determine kinetic parameters of the interaction of emerin with full-length β-DG or the TM domain of β-DG alone. GST–β-DG (**A**), GST–TMβ-DG (**B**), GST–emerin (**C**) of GST alone (**D**) were layered onto an SPR sensor, previously coated with GST–emerin. The responses (µRIU) observed for each protein concentration at different incubation times are shown. Analysis of emerin self-assembly served as positive control, while its interaction with GST alone served as negative control; (**E**) The kinetic parameters of the interaction of emerin with β-DG, TM of β-DG or emerin are shown.

To further characterize the β-DG–emerin interaction, surface plasmon resonance (SPR) was used to measure association and dissociation constants and equilibrium affinities for β-DG–emerin binding. In the experimental design adopted, recombinant GST–emerin was immobilized onto polyethylene glycol/carboxyl sensor slides, and subsequently, different concentrations of recombinant proteins GST–β-DG or GST–TM-β-DG (soluble analytes) were injected onto the coated surface. Since self-assembly of emerin was previously reported [18], the intermolecular association of emerin was analyzed as positive control. Emerin bound itself with high affinity, 1.87 nM (Figure 3C), which is line the emerin–emerin affinity reported in *C. elegans* (14 nM) [19]. On the other hand, analysis of the interaction between emerin and GST alone, served as negative control and accordingly no interaction between these proteins was detected (Figure 3D). Remarkably, emerin was found to bind with high affinity to full-length β-DG (3.32 nM) and to its TM domain (25.3 nM) (Figure 3A,B). Although both full-length and the TM domain of β-DG were associated with emerin with KD values in the nanomolar range, the slight difference in the observed KDs is in line with was observed in the IP assays, in which endogenous emerin was pulled down by GFP–full-length-β-DG, but only to a lesser extent by GFP–TMβ-DG (see above). In fact, the association constants between emerin and either full-length or TM domain of β-DG were different, while their dissociation constants were quite similar (Figure 3E). Overall, these data suggest that in vivo the interaction between the TM domains of emerin and β-DG may be strengthened by some contribution offered by other segments of the proteins.

**Figure 4:**
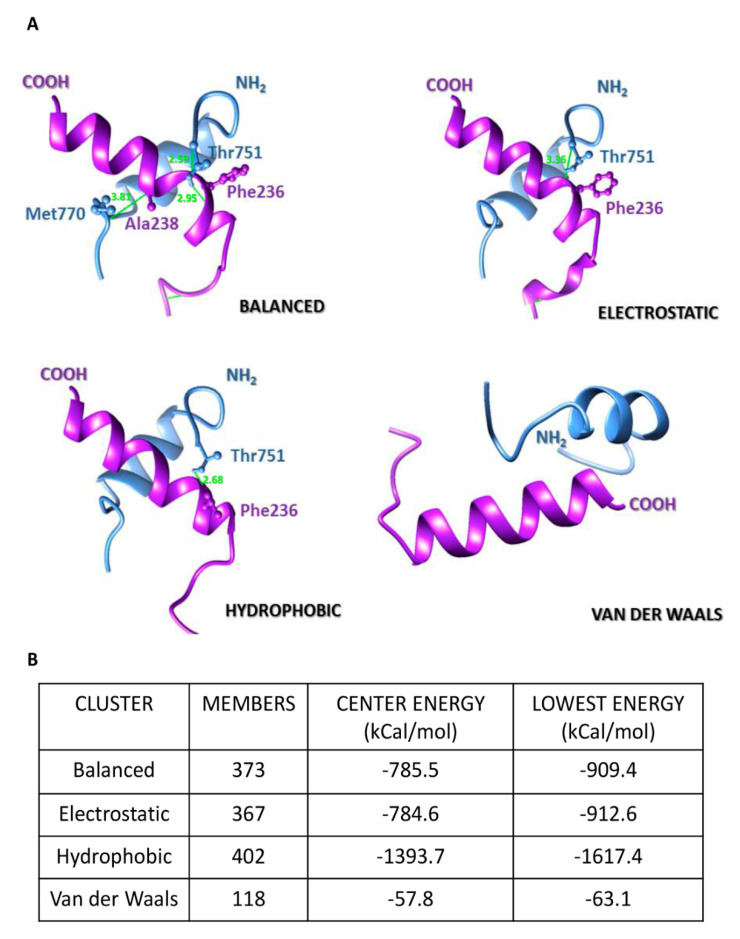
In silico analyses of emerin–TM-β-DGTM interaction mode. (**A**) Docking of the TM domains of emerin (purple) and β-DG (blue) and their modes of interaction. The C-terminal region of emerin and the N-terminal region of β-DG at the outer nuclear membrane are indicated. Residues involved in clashes/contacts are displayed in ball-and-stick conformation and colored accordingly to its backbone structure, with clashes/contacts displayed as green lines (distance ≤ 4.0 Å); (**B**) Conformer members of each interactive cluster and their corresponding values of energy (in kcal/mol) at center of complexes and for the most stable state (lowest energy) between the TM domains are shown.

To predict the mode (s) of interaction between the TM domains of β-DG and emerin, we performed a molecular docking analysis. De novo 3D-models of β-DG and emerin, generated in the absence of crystal structures of reference, were not of sufficient quality (Supplementary Figure S2) (Ramachandran plot analysis showed 687/893 and 213/259 residues in allowed areas, respectively, as calculated by MolProbity server). To avoid structure-biased predictions, only the TM regions of β-DG and emerin were modeled by the I-Tasser server (19/23 and 20/22 residues in allowed areas, respectively) and included in open docking analyses using ClusPro v2.0 platform, which provides predictions in balanced, electrostatic, hydrophobic and Van der Waals conformations. Figure 4 shows top models suggesting that the TM domains intersect in a crossed mode, as supported by balanced (total sum of attractive and repulsive energies), electrostatic and hydrophobic complex models (Figure 4A), while Van der Waals forces did not virtually contribute to TM–TM interactions (−63 kcal/mol, Figure 4B). In the balanced complex, clashes/contacts (green lines) between the TM domains involve hydrogen G1 and backbone of Thr751 as well as backbone of Met770 of β-DG, with the backbones of Phe236 and Ala238 of emerin, while Thr751–Phe236 contact predominates in both the electrostatic- and the hydrophobic-favored complexes. Considering the hydrophobic environment at the inner nuclear membrane, the lowest-energy complex (i.e., highest stability), corresponds to the hydrophobic-favored complex (−1617 kcal/mol, Figure 4B). Thus, it is likely that a limited set of amino acid residues participate in clashes/contacts between the TM domains of β-D and emerin, through predominant hydrophobic and contributing electrostatic forces.

### 2.2. Localization of β-DG at the NE Depends on the Emerin TM Domain

**Figure 5:**
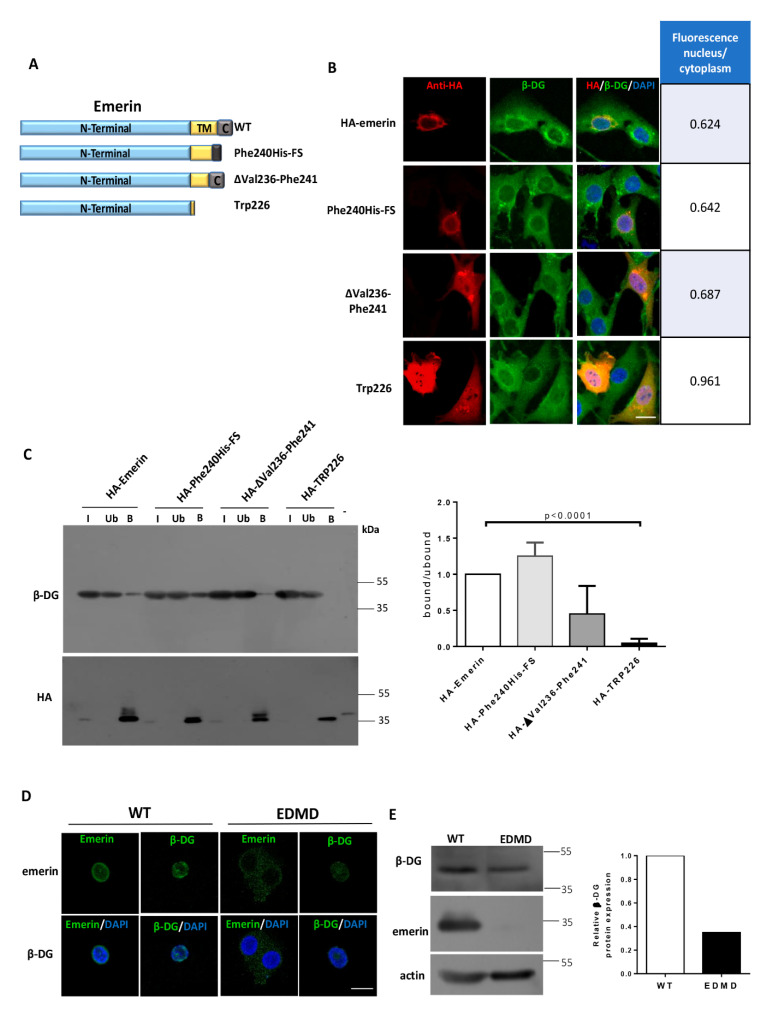
Effect of emerin mutants on the subcellular localization of β-DG. (**A**) Schematic representation of emerin and the emerin mutants Phe240His–FS, ΔVal236–Phe241 and Trp226, (**B**) C2C12 cells grown on coverslips were transiently transfected to express the indicated HA-tagged emerin constructs. At 24-post-transfection, the cells were fixed, double immunostained with anti-HA and anti-β-DG antibodies and counterstained with DAPI to visualize nuclei. Typical single optical Z-sections obtained by CLSM are shown, with arrows indicating subcellular localization of β-DG in transfected cells (scale bar 10 µm). The nuclear to cytoplasmic fluorescence ratio (Fn/c) of β-DG was quantified in the cells transfected with HA-tagged emerin proteins (right chart); (**C**) Interaction of β-DG with HA-tagged emerin proteins was analyzed by immunoprecipitation using anti-HA antibodies. Bound (B) and unbound (Ub) fractions were analyzed by western blotting using specific antibodies against β-DG and HA. Input corresponds to 5% of total lysates prior to immunoprecipitation. Right. Densitometric analysis of immunoblot autoradiograms was carried out to estimate the relative binding ability of HA-tagged emerin proteins to endogenous β-DG (the band intensity of β-DG in the bound fraction was divided by the band intensity of β-DG in the unbound fraction, and bound/unbound ratio of WT emerin was set at 1. Data correspond to the mean ± SD from three separate experiment, with significant differences calculated by unpaired *t*-test; (**D**) Distribution of β-DG in B-lymphocyte cultures derived from a patient with EDMD or from healthy individual. B-lymphocytes grown on coverslips were immunostained for emerin or β-DG and counterstained with DAPI to decorate nuclei. Representative single optical Z-sections obtained by CLSM from two independent experiments are shown (scale bar 10 µm). Right; (**E**) Lysates from EDMD and control B-lymphocytes were analyzed using antibodies against emerin, β-DG and actin (loading control). Quantification of β-DG relative expression from two independent experiments is shown (right graph).

To ascertain whether targeting of β-DG to the NE requires the transmembrane domain of emerin, we evaluated the effect of human emerin TM mutations, which occur in EDMD patients, on β-DG nuclear distribution (Figure 5A). The missense mutation Phe240His–FS and the deletion ΔVal236–Phe241 result in shorter hydrophobic transmembrane domains of emerin, while a mutation introducing a frame shift at Trp226 virtually eliminates its TM domain impairing targeting to the inner nuclear membrane [17]. C2C12 myoblasts overexpressing HA-tagged WT emerin exhibited a predominant cytoplasmic distribution of endogenous β-DG, with a clear localization at the NE and virtually no signal in the nucleoplasm, as observed in a middle Z-section (Figure 5B). This distribution pattern of β-DG remained substantially unchanged in cells overexpressing Phe240His–FS and ΔVal236–Phe241 emerin mutants; instead, overexpression of the mutant with the stop codon at Trp226 resulted in increased nucleoplasmic labeling of β-DG (Figure 5B), as shown by its fluorescence nucleus/cytoplasm ratio (right chart). We next investigated whether the mutations in the TM preclude emerin interaction with β-DG by immunoprecipitation assays. Both Phe240His–FS and ΔVal236–Phe241 mutants maintained the ability to interact with endogenous β-DG, as observed with WT emerin. In contrast, the Trp226 mutant was unable to bind to endogenous β-DG (Figure 5C). Finally, we examined the distribution of β-DG in a B-lymphocyte cell line derived from a patient with EDMD. These EDMD B-lymphocytes harbor a five base pairs insertion in exon 6 of emerin gene, which in turn results in a stop codon at amino acid 238. The mutated emerin therefore lacks 6 amino acid residues of the TM domain and the entire C-terminal domain. Immunostaining of emerin at the nuclear periphery was significantly decreased in EDMD B-lymphocytes, and virtually no emerin was detected by western blotting, compared with B-lymphocytes derived from a healthy subject (Figure 5D). Interestingly, the loss of emerin resulted in a decrease of both immunolabeling and protein levels of β-DG, compared with control B-lymphocytes (Figure 5D). Collectively these data imply that localization of β-DG at the NE depends on the TM of emerin, which is required to have sufficient length and degree of hydrophobicity to support the interaction between the two proteins.

### 2.3. The Loss of Dystroglycan Impairs Emerin Stability and Function

**Figure 6:**
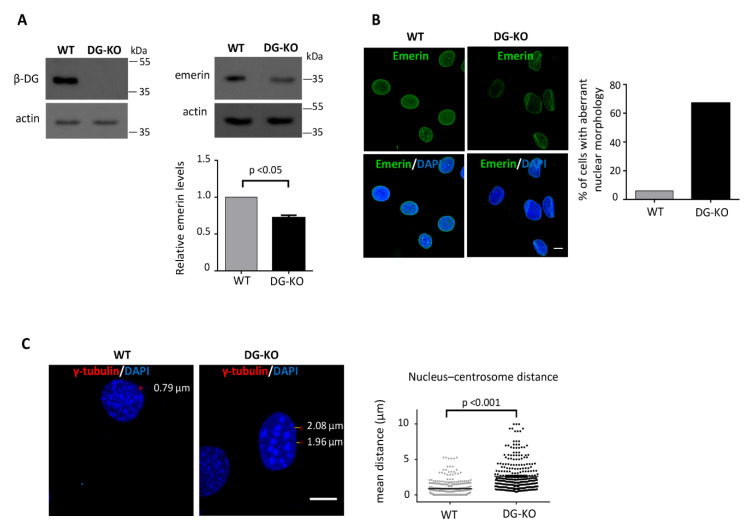
DG–KO cells showed decreased emerin levels, aberrant nuclear morphology and increased nucleus-centrosome distance. (**A**) Emerin levels were assessed in WT and DG–KO C2C12 cells by western blotting, using antibodies against β-DG, emerin and actin (loading control). Results correspond to mean +SEM of three separate experiments, with a p-value showing significant differences (unpaired *t*-test); (**B**) WT and DG–KO C2C12 cells were immunolabeled for emerin and counterstained with DAPI to decorate nuclei. The percentage of cells with aberrant nuclear morphology was calculated from three independent experiments (*n* = 150 nuclei), with a p-value showing significant differences (unpaired *t*-test). Scale bar, 10 µm; (**C**) WT and DG–KO C2C12 cells were double stained with anti-γ-tubulin antibodies and DAPI to decorate centrosomes and nuclei, respectively. Typical nuclei showing centrosome positioning are shown; scale bar, 10 µm. Nucleus-centrosome distance were measured in overlaid images using Leica Application Suite, Advanced Fluorescence Lite imaging processing software. Data in the graph correspond to the mean ± SD of triplicate experiments (*n* = 300 nuclei), with p-value denoting significant difference (student’s *t*-test).

To determine the biologic significance of emerin–β-DG interaction, the impact of the absence of DG on emerin levels/function was analyzed using C2C12 DG–KO cells. The emerin levels were found to decrease by 40% in DG–KO cells (Figure 6A). Therefore, we next ascertained whether the lack of DG compromises emerin function by evaluating two cellular processes that depend on emerin, namely nuclear morphology [20] and the linkage of centrosomes to the outer nuclear membrane and anchorage of centrosomes to the NE [21]. Interestingly, immunolabeling for emerin revealed prominent nuclear morphology deformities in 35% of DG–KO cells (Figure 6B), including fissured, kidney-shaped and blebbed nuclei. Nuclear defects were scored at multiple passage numbers (pp. 11–14) and were significantly different at all stages, suggesting that they were not due to culture artifacts. To determine centrosome position relative to the nucleus, cells were stained for γ-tubulin and DAPI to decorate γ-tubulin ring complex and nuclei, respectively. In WT cells, centrosomes were located juxtaposed of the nucleus with an average value of 0.88 μm, while DG–KO cells exhibited increased centrosome-nucleus distance with an average value of 2.62 μm (Figure 6C).

**Figure 7:**
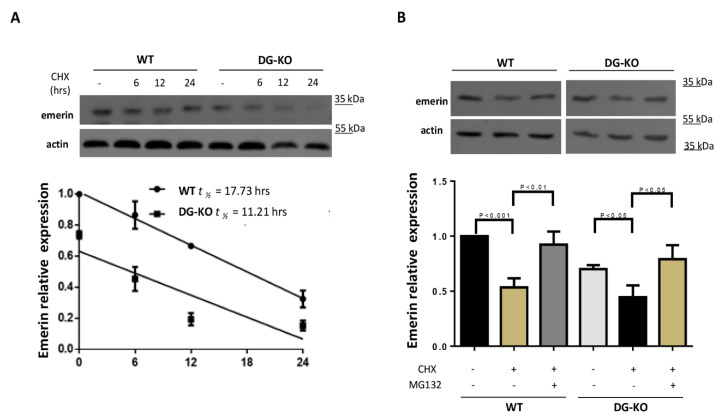
Loss of β-DG accelerates emerin degradation by the proteasome. (**A**) WT and DG–KO C2C12 myoblasts were treated with cycloheximide (CHX) for the indicated time intervals. Cell lysates were then subjected to western blot analysis using specific antibodies for emerin and actin (loading control). Bottom panel. Emerin half-life (t1/2) was calculated by densitometry analysis of western blots, as described in Methods. Data correspond to mean +SEM of three separate experiments, and the linear regression plot was obtained using Graphpad Prism 6 software; (**B**) WT and DG–KO C2C12 myoblasts treated with CHX as in (**A**) were further incubated for 24 h with MG132 (proteasome inhibitor) or vehicle alone (-). Data correspond to mean +SEM of three separate experiments, with p-values indicating significance differences (unpaired *t*-test).

We envisaged that decreased emerin levels exhibited by DG–KO cells may be the result of a reduction in protein stability, due to the lack of its interacting partner, β-DG. WT and DG–KO cells were treated with cycloheximide to inhibit protein synthesis, and emerin turnover was then analyzed. A reduction in the half-life of emerin from 17.7 to 12.2 h was found in DG KO cells (Figure 7A) and this decrease was reversed upon treatment with the proteasome inhibitor MG132 (Figure 7B), which is consistent with the idea that degradation of emerin is increased in cells lacking DG.

## 3. Discussion

The nuclear envelope (NE) is a complex membrane barrier that functionally separates the nucleus from the cytoplasm and orchestrates a variety of cellular processes, including nuclear architecture, DNA replication, gene expression and chromatin organization. The essential role of the NE is highlighted by the existence of a growing number of human diseases caused by mutations in genes encoding NE components, known collectively as laminopathies [1]. Considering the functional diversity of the NE it is likely that new NE-located proteins must still be identified. Our recent studies revealed that β-DG is a previously unrecognized NE component, which is likely to be involved in the maintenance of nuclear structure and function, probably by serving as a scaffold for the assembly of a NE protein complex composed of emerin and lamins A/C and B1 [4,10]. Thus, characterization of the molecular interaction of β-DG with NE proteins would help to decipher its function in the nucleus. In this study, we describe for the first time some molecular details underlying the interaction between β-DG and emerin and provide compelling evidence showing the biologic significance of this interplay. We focused on β-DG–emerin association because emerin is a major NE protein that modulates diverse cellular processes [12,13] in fact, emerin deficiency, due to mutations in its encoding gene, result in EDMD type 1, a myopathy characterized by progressive muscle degeneration and weakness and usually cardiac problems [22].

We determined that these two proteins associate directly in vitro by pull-down assays using recombinantly expressed and purified truncated variants of both β-DG and emerin, that these two proteins associate directly in vitro, with the interaction being mediated, at least in part, by their respective TM domains. Binding of β-DG with emerin occurs in the inner nuclear membrane environment, as the TM of β-DG fused to GFP co-immunoprecipitated in intact cells with endogenous emerin. We propose that β-DG (a type I transmembrane protein) associates with emerin (a type II transmembrane protein) by a lateral and antiparallel TM helix–helix interaction, as many single transmembrane span proteins do, including integrins and receptor-linked tyrosine kinases; transmembrane (TM) helices of integral membrane proteins allow strong and specific noncovalent protein–protein interactions [23]. Supporting this idea, emerin bound in vitro with high affinity to the TM of β-DG (KD value of 25.3 mM), as shown by SPR assays. This affinity value is similar to those obtained for other emerin interactions, including BAF-1-emerin (57 nM), emerin–emerin (14 nM) [19] and emerin-lamin A (40 nM) [24]. Furthermore, protein docking of the TM domains of β-DG and emerin predicted a crossed-type of interaction between these domains, with only few residues involved in the lateral helix-helix contacts, where predominant hydrophobic and contributing electrostatic forces are implicated. We hypothesize that this nonparallel reciprocal positioning would allow the reciprocal intra- and extra-nuclear domains of these protein to be at closer proximity; in fact, the presence of other interactions not depending on the TM domains cannot be ruled out. It is worth noting that currently there is no knowledge about the factors regulating association between the TM domains of NE proteins. Therefore, further experiments using TOXCAT assays, a system, designed to analyze transmembrane α-helices association in a biologic membrane [25], as well as site-specific mutagenesis against key residues on the TM of each protein suggested by docking prediction, would aid to define biochemical properties of the TM–TM interaction between β-DG and emerin.

Interaction with multiple partners confers on emerin a multifunctional character: emerin regulates gene expression via its association with both Lim-domain-only 7 (Lmo7; a myogenic transcription factor) [26,27] and Germ cell-less (GCL; transcription repressor involved in cell proliferation) [24]. Emerin is also involved in cell signaling through its interaction with the Wnt pathway component β-catenin [28,29,30]. On the other hand, emerin preserves nuclear architecture by its association with lamin A/C [31] and regulates post-mitotic nuclear assembly and chromatin architecture in conjunction with Barrier-to-autointegration factor (BAF/Banf1) and histone deacetylase 3 (HDAC3), respectively [16,32]. Thus, it is plausible to propose that binding to β-DG modulates emerin function. To address the physiological significance of this interaction, we evaluated how the loss of DG impact on emerin function, using DG–KO C2C12 cells with no expression of DG (α-DG and β-DG) [33] The lack of DG resulted in a 30% decrease in emerin levels, due to its accelerated turnover in DG–KO cells. Decreased emerin levels were reversed by treatment with MGG132, (proteasome specific inhibitor), which implies that the lack of a NE partner (β-DG), targets emerin to proteasomal degradation. The recovery of emerin protein level by reexpressing β-DG in DG-K0 cells is required to confirm this hypothesis. Previous studies showed that emerin turnover is mediated by the proteasomal pathway [34]. Emerin deficiency of DG–KO cells in turn causes alterations in emerin-dependent processes, including nuclear morphology maintenance and anchorage of centrosomes to the NE. Although CRISPR–Cas9 genome editing ablates the expression of both α-DG and β-DG [33], we believe that emerin dysfunction of DG–KO cells is mechanistically linked to the nuclear deficiency of β-DG, because emerin is a β-DG interacting partner, as shown herein. Nonetheless, the possibility that the lack of α-DG affects emerin by perturbing the signaling across the plasma membrane-cytoskeleton-nucleus axis cannot be ruled out. Targeting of β-DG to the NE seems strictly to require the TM of emerin, as overexpression of an emerin nonsense mutant, which lacks almost all of its TM domain (Trp226), resulted in mislocalization of β-DG from the NE to the nucleoplasm, because the Trp226 mutant seems unable to interact with endogenous β-DG.

Collectively our data are consistent with the idea of β-DG being a modulator of emerin function. Therefore, we hypothesize that disruption of β-DG–emerin interaction may play a role in the pathogenesis of EDMD. Supporting this notion, DG–KO C2C12 cells recreate distinctive features of EDMD cells, such as aberrant nuclear morphology (as mentioned above) and impaired myogenic differentiation (unpublished data). Furthermore, B-lymphocyte cell cultures derived from a patient with EDMD showed decreased protein level of β-DG. We speculate that deficiency of β-DG may lead by itself to some of the observed EDMD phenotype, as over 60% of EDMD patients remain to be associated with a genetic defect [35].

## 4. Materials and Methods

### 4.1. Cell Culturing and Transfection

Mouse myoblasts C2C12 (ATCC^®^ CRL-1772™) were cultured in Dulbecco’s modified Eagle’s medium (DMEM) (Invitrogen, Carlsbad, CA, USA) supplemented with 10% (*v/v*) fetal bovine serum, 50 U/mL penicillin, 50-µg/mL streptomycin and 1-mM sodium pyruvate at 37 °C, in a humidified 5% CO2 cell incubator, as previously [36], while generation and characterization of C2C12-derivative DG knockout cell line (DG–KO) was previously published [33]. Where appropriate, cells were transfected using Lipofectamine 2000 (Invitrogen, Carlsbad, CA, USA), following the manufacturer’s protocol. For indirect immunofluorescence assays, 2 µg of plasmid DNA in 100 mL serum-free DMEM was mixed with 2 µL Lipofectamine 2000 and incubated for 30 min at room temperature. The transfection mixture was then combined with 2 mL fresh medium and added to cells grown on coverslips at 75% confluence. For western blotting, cells seeded on 60 mm wells were transfected using 4 µg of plasmid DNA and 8 µl Lipofectamine 2000, as described above. Cells were processed 24 h post-transfection. Where indicated, cells were incubated with 30-µg/mL cycloheximide (CHX; Sigma-Aldrich, St. Louis, MO, USA) to inhibit protein synthesis and then harvested at 0, 6, 12 and 24 h of CHX treatment for further analysis. The proteasome inhibitor MG132 (Sigma-Aldrich, St. Louis, MO, USA), was used at 10 µM for 24 h. B-lymphocyte cell lines from a patient with EDMD (GM16864) or from a healthy individual (AG10097) were purchased from Coriell Cell Repositories (CCR) (Camden, NJ, USA) and cultured in RPMI medium.

### 4.2. Plasmid Constructs and Antibodies

To construct vector pSG5–emerin, human emerin cDNA was amplified by PCR from HeLa cells total RNA, using an M–MLV reverse transcriptase coupled to a high fidelity polymerase, Pfu turbo (Agilent Technologies, Inc., Santa Clara, CA, USA) and the primers described in Table 1 that contained BamHI restriction sites. PCR product was digested with BamHI and cloned in frame into pSG5 vector (Agilent Technologies, Inc., Santa Clara, CA, USA), previously dephosphorylated and digested with BamHI. For construction of vector pSG5–β-DG, human β-DG cDNA was amplified by PCR using pGEX4TI-β-DG as the template [16] and the primers shown in Table 1 that contained EcoRI restriction sites. PCR fragment was then digested with EcoRI and inserted in frame into pSG5 vector, previously dephosphorylated and digested with EcoRI.

To construct GST-tagged β-DG plasmids, cDNA fragments corresponding to the different domains of β-DG were amplified by PCR with a high fidelity Taq polymerase, platinium (Invitrogen, Carlsbad, CA, USA), using the corresponding oligonucleotides shown in Table 1 that contained EcoRI restriction sites and pSG5–β-DG as the template. Each PCR fragments was then cloned in frame into pCR2.1–TOPO vector (Invitrogen, Carlsbad, CA, USA) to be further cleaved by EcoRI restriction and cloned in frame into pGEX-4T1 (Amersham Bioscience, GE Healthcare, Buckinghamshire, UK), previously dephosphorylated and digested with EcoRI. To construct GST-tagged emerin plasmids named pGST–NT and pGST–CT, cDNA fragments corresponding to these domains were PCR-amplified with Taq polymerase, Platinium (Invitrogen, Carlsbad, CA, USA), using the corresponding oligonucleotides shown in Table 1, which contained EcoRI restriction sites and pSG5–emerin as the template. PCR fragments were further cloned in pCR2.1–TOPO vector to be further cleaved by EcoRI restriction and cloned in frame into pGEX-4T1 (Amersham Bioscience, GE Healthcare, Buckinghamshire, UK), previously dephosphorylated and digested with EcoRI. Finally, pGST–TM vector expressing the TM of emerin was engineering following the same strategy, using the appropriate oligonucleotides that contained BamHI restriction (Table 1). For construction of GFP-tagged plasmids, cDNA fragments corresponding to the transmembrane domains of β-DG (amino acids 751–774) and emerin (220–243 amino acids) were amplified by PCR, using the primers described in Table 1 and vectors pSG5–β-DG and pSG5–emerin as the templates, respectively and further cloned into pCR2.1–TOPO. Then, BglII-EcoRI restriction fragments released from pCR2.1–TOPO were cloned in frame into BglII-EcoRI digested pEGFP-C1 vector Addgene (Watertown, MA, USA), to generate vectors pGFP–TMβ-DG and pGFP–TMβ–Eme.

The following primary antibodies were used: mouse monoclonal antibodies directed against β-DG (MANDAG2) [16], Υ-tubulin (T6557) (Sigma-Aldrich, St. Louis, MO, USA), influenza hemagglutinin (HA; sc-7392) and GST (B-14) (Santa Cruz Biotechnology, CA, USA); rabbit polyclonal antibodies directed against GFP (sc-8334), emerin (sc-15,338) (Santa Cruz Biotechnology, CA, USA), HA (71–5500) (Invitrogen, Carlsbad, CA, USA) and actin (a gift from Dr. Manuel Hernández, CINVESTAV-IPN, Mexico).

### 4.3. In Vitro Transcription/Translation and GST Pull-Down Assay

In vitro binding of β-DG with emerin was analyzed by GST-based binding assays. Emerin and β-DG proteins were synthetized in vitro using pSG5–emerin and pSG5–β-DG plasmids and the TNT^®^ Quick Coupled Transcription/Translation System (Promega Corporation, Madison, WI, USA) in the presence of [S35]-methionine (Amersham Bioscience, GE Healthcare, Buckinghamshire. GST and GST-tagged fusion proteins were expressed and purified from BL21 E.coli bacteria under native conditions, as previously reported [37]. Affinity purified GST and GST-tagged β-DG proteins immobilized on glutathione–sepharose beads (2.5 μg) were incubated with 10 µL of [S35]-emerin overnight at 4 °C, with agitation in 150 µL of binding buffer (15-mM HEPES pH 7.1, 140-mM K-acetate, 5-mM MgSO_4_). Likewise, GST and GST-tagged emerin proteins were incubated with [S35]-β-DG. Thereafter, glutathione beads were recovered by centrifugation at 1000× *g* at 4 °C and washed three times in 1 mL of ice-cold washing buffer [50-mM Tris-HCl pH 7.5, 150-mM NaCl, 1-mM EDTA pH 8.0, 1-mM PMSF, 1% (*v/v*) Triton X-100], and then, bound radioactive proteins were mixed (1:1) with 2X Lemmli loading buffer (4% SDS, 20% glycerol 125-mM Tris, pH 6.8, 0.02% Bromophenol blue 200-mM DTT) and further separated by 10% SDS–polyacrylamide gels. Gels were fixed 1 h with fixing buffer (7% acetic acid, 40% methanol), dried, exposed on a phosphor screen and imaged using the Typhoon trio (Amersham Bioscience, GE Healthcare, Buckinghamshire, UK).

### 4.4. Indirect Immunofluorescence and Confocal Microscopy Analysis

Cells cultured on coverslips were fixed for 10 min with 4% paraformaldehyde, permeabilized for 10 min by exposure to 0.2% Triton X-100 in PBS and blocked for 20 min with 0.5% gelatin in PBS at room temperature, prior to incubation overnight at 4 °C with appropriate primary antibodies and then with the appropriate secondary fluorochrome-conjugated antibodies (Jackson ImmunoResearch Laboratories, West Grove, PA, USA). Double immunostaining was carried out by incubation overnight at 4 °C with the second primary antibody and the next day with the corresponding secondary antibody. Cells were incubated for 10 min at room temperature in PBS with 1-mg/mL diamidino-2-phenylindole (DAPI) (Sigma-Aldrich, St. Louis, MO, USA) to stain nuclei. After washing, coverslips were mounted on microscope slides with VectaShield (Vector Laboratories, Inc., Burlingame, CA, USA) and examined on a confocal laser scanning microscope (TCP-SP5, Leica, Heidelberg, Germany) employing a Plan Neo Fluor 63× (NA = 1.4) oil-immersion objective. Manders overlap coefficients were calculated for double labeling immunofluorescences, using red (Alexa 594 nm) and green (FITC 488 nm) channels and the ImageJ plugin JACoB. Quantitative analysis to determine the nuclear to cytoplasmic ratio of fluorescence (Fn/c) was carried out using Image J software.

### 4.5. Western Blotting

Cells were centrifuged, washed with PBS pH 7.4 and resuspended in 150 μL lysis buffer (50 mM Tris-HCl pH 8.0, 150 mM NaCl, 1 mM PMSF, 1% Triton X100, 0.1% SDS, 0.5% sodium deoxycholate, 1X complete protease inhibitors cocktail and 1 mM PMSF), homogenates were then clarified by centrifugation at 4 °C and protein concentration determined by the Bradford method. The suspension was then sonicated at 3.5 microns on Soniprep 150, applying 3 bursts of 15 s each, with 30 s interval between each sonication. Lysate aliquots (40–80 μg of protein) were mixed (1:1) with 2X Laemmli buffer (4% SDS, 10% 2-mercaptoethanol, 20% glycerol, 0.004% bromophenol blue and 0.125-M Tris-HCl pH 6.8), prior to be electrophoresed on 10% SDS–polyacrylamide gels, using the electrophoresis running buffer (25-mM Tris base, 250-mM glycine pH 6.8 and 0 0.1% SDS). Proteins were then transferred to nitrocellulose membranes (Hybond-N+, Amersham Pharmacia, GE Healthcare, Buckinghamshire, UK) on Transblot apparatus (Bio-Rad, Hercules, CA, USA), using the transfer buffer (39-mM glycine, 48-mM Tris base, 0.037% SDS, 20% methanol). Membranes were then blocked for 1 h at room temperature in agitation with TBS-T (100-mM Tris-HCl pH 8.0, 150-mM NaCl, 0.5% [*v/v*] Tween-20) and 6–15% [*w/v*] low-fat dried milk) and further incubated overnight at 4 °C in agitation with the appropriate primary antibody. After three washes in TBS-T, the membranes were incubated with the appropriate horseradish peroxidase–conjugate secondary antibody (Amersham Pharmacia, GE Healthcare, Buckinghamshire, UK) and developed in X-ray films (Carestream Medical X-ray Blue/MXB Film), using ECL western blotting analysis system (Amersham Pharmacia, GE Healthcare, Buckinghamshire, UK), according to the manufacturer’s instructions. Blot images were acquired with the Gel Doc EZ System (Bio-Rad Laboratories, Inc., Berkeley, CA, USA) and subjected to densitometric analysis using Image Lab 6.0.1 software (Bio-Rad Laboratories, Inc., Berkeley, CA, USA). To normalize protein expression, the band intensity of the target protein was divided by the band intensity of the loading protein (actin). To calculate emerin half-life, the emerin protein level was assessed by densitometry analysis of autoradiograms, during a time course of cycloheximide treatment. The band pixel intensity of emerin was divided by the band pixel intensity of actin (loading control) for each lane and the normalized emerin level present in WT C2C12 myoblasts at t0 was set at 1.0. Normalized emerin values were fit to exponential decay curves to calculate protein half-life and the linear regression plot was obtained using Graphpad Prism 6 software (www.graphpad.com).

### 4.6. Immunoprecipitation

Recombinant protein G-agarose beads (Invitrogen, Carlsbad, CA, USA) were equilibrated with gentle agitation for 4 h in lysis buffer and then C2C12 whole cell extracts (1 mg) incubated with the beads for 2 h at 4 °C, followed by incubation of the cleared extracts overnight at 4 °C with 5 μg of anti-hemagglutinin (HA) immunoprecipitating antibodies. As a negative control, parallel incubations with an irrelevant IgG antibody were performed. After that, equilibrated protein G-agarose beads blocked previously with 4% BSA were added to the protein extracts and incubated overnight at 4 °C. Immune complexes were collected by centrifugation at 1250× *g* for 5 min, washed 3 times for 10 min with RIPA buffer (50-mM Tris-HCl pH 8.0, 300-mM NaCl, 0.1% (*v/v*)), 1X complete protease inhibitor tablet, 0.5% (*v/v*) Triton X-100 and 0.5-mM PMSF and precipitated proteins separated by SDS-PAGE for western blotting analysis. Immunoprecipitation using the GFP-Trap^®^ bead system (ChromoTek, Munich, Germany) was performed in accordance with the manufacturer’s instructions.

### 4.7. Surface Plasmon Resonance

Kinetics analyses were performed in a Reichert (Depew, New York, NY, USA) SR7500DC dual-channel SPR instrument. For analysis of the kinetics of emerin–β-DG and emerin–TM of β-DG interactions, planar polyethylene glycol/carboxyl sensor slides were used. The flow rate for all steps was determined with phosphate-buffered saline with Tween-20 (PBST) at 40 µL/min. GST–emerin was coupled to the sensor by passing N-ethyl-N′-(3-dimethylaminopropyl) carbodiimide–N-hydroxy-succinamide (EDC–NHS) for 5 min, immobilizing GST–emerin in 20-mM sodium acetate buffer (pH 4.0) at 12.5 µg/mL just over the left channel for 8 min and blocking with 1-M ethanolamine (ETN) for 10 min. The kinetic analysis with the ligands (GST–β-DG, GST–TMB–DG, GST–emerin and GST alone as negative control) were done by injecting five increasing concentrations of ligand in PBST in triplicates for 150 s to see association phase, after that, PBST was load for 150 s to see the dissociation phase. Between each one of triplicate experiments, PBST was perfused for 15 min to achieve a new baseline before the next injection, using several injections from lower to higher concentration of the analyte without regeneration [38,39]. Data were recorded using Integrated SPRAutolink software and BioLogic scrubber 2 software (Campbell, Australia) was used for curve fitting and data analysis. Statistical analyses (One-way ANOVA, Tukey’s multiple comparisons test) were performed with GraphPad Prism 6.0 (www.graphpad.com) using the values from 30 different injections. *p*-values of <0.05 were considered statistically significant. The data were expressed as means ± standard errors of the means.

### 4.8. In Silico Analyses of Emerin-β-DG Interaction

In initial analyses, protein structure models of emerin (259 amino acids long) and β-DG (893 amino acids long) were carried out by open modeling using I-Tasser server (http://zhanglab.ccmb.med.umich.edu/I-TASSER/) [40] and its stereochemical quality was assessed using the MolProbity server (http://molprobity.biochem.duke.edu/). Further, the structure models of helix-rich TM domains (residues 225–247 in emerin and 551–773 in β-DG) were raised from homology modeling with I-Tasser server. The resultant structures were subjected to protein–protein docking using the ClusPro 2.0 server (https://cluspro.bu.edu/signup.php) [41], which rendered sets of balanced, electrostatic-favored, hydrophobic-favored and Van der Waals + electrostatic-favored clusters. Top predictions were considered for further visualization and editing using UCSF Chimera v10.1.1 software, where distances in clashes/contacts were set at ≤ 4.0 Å.

## 5. Conclusions

In summary, we showed that binding of β-DG with emerin takes place at least in part, through the association of their corresponding TM domains. This interaction is biologically relevant because β-DG confers stability on emerin and positively regulates its function; reciprocally, emerin is required for β-DG to be targeted to the NE. The potential involvement of β-DG in the phenotypes observed in patients affected by EDMD deserves further investigation.

## Figures and Tables

**Table 1:** Plasmids used in this study.

Plasmids	Oligonucleotide Sequences	Reference/Source
pSG5 plasmids		
pSG5–emerin	Forward 5′-ACTGGATCCATGGACAACTACGCAGATCT-3′	This study
	Reverse 5′-ACTGGATCCCTAGAAGGGGTTGCCTT-3′,	
pSG5–β-DG	Forward 5′-CCGAATTCATGTCCATCGTGGT-3′	This study
	Reverse 5′-CCGAATTCGATTAAGGTGGGACATA-3′	
GST-tagged β-DG plasmids		
GST–β-DG		[14]
GST-CT	Forward 5′-GCATGAATTCTACCGCAAGAAGCGGAAGG-3′	This study
	Reverse 5′-GCATGAATTCTTAAGGTGGGACATAGGGAG-3′	
GST–NT+TM	Forward 5′-GCATGAATTCTCCATCGTGGTGGAATGGAC-3′	This study
	Reverse 5′-GCATGAATTCTTAGCAGATCATGGCAATGATGC-3′	
GST–NT	Forward 5′-CTGAATTCTCCATCGTGGTGGAA-3′	This study
	Reverse 5′-CTGAATTCTTACAGGTAGACATCAT-3′	
GST–TM	Forward 5′-CTGAATTCCACACAGTCATTCC-3′	This study
	Reverse 5′-CTGAATTCTTAGCAGATCATGGCA-3′	
GST-tagged emerin plasmids		
pGST–emerin		[15]
pGST–NT	Forward 5′-ACTGGATCCATGGACAACTACGCAGATCT-3′	This study
	Reverse 5′-CTGGATCCCTAATCCTGGCCCA-3′	
pGST–TM	Forward 5′-CTGGATCCCGCCAGGTCCCG-3′	This study
	Reverse 5′-CTGGATCCCTAGTGGTAAATGAA-3′	
pGST–CT		[15]
GFP-tagged plasmids		
GFP–β-DG		[9]
GFP–emerin		[16]
GFP–TMβ-DG	Forward 5′-CTAGGATCCCACACAGTCATTCCG-3′	This study
	Reverse 5′-CTCGGATCCTTAAAGGGTAAGCTTGC-3′	
GFP–TM–Eme	Forward 5′-ATGGATCCCGTGCTCCTGGGGCT-3′	This study
	Reverse 5′-ATGGATCCCTAGTGGTAAATGAAGAAG-3′	
HA-tagged plasmids		
pHA–emerin		[17]
pTrp226		[17]
pΔVal236–Phe241		[17]
pPhe240His–FS		[17]

## Supplementary Materials

Supplementary materials can be found at https://www.mdpi.com/1422-0067/21/17/5944/s1. Figure S1. In vitro synthesis of ^35^S-labeled emerin and β-DG using a coupled transcription/translation system. Figure S2. Docking of full-length emerin and β-DG protein structures.
