# Supplementary material for: The Molecular Basis and Biologic Significance of the β-Dystroglycan-Emerin Interaction
Source: Int J Mol Sci. 2020 Aug 19;21(17):5944. doi: 10.3390/ijms21175944 (PMC7504044; doi:10.3390/ijms21175944)
Supplement: Supplementary file 1 [file ijms-21-05944-s001.pdf]

Supplementary Figure 1

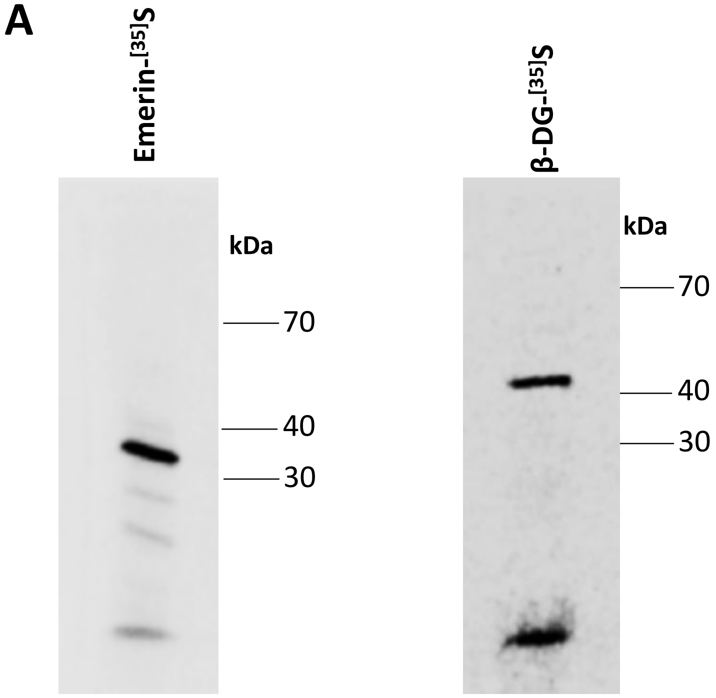

Supplementary Figure 2

A

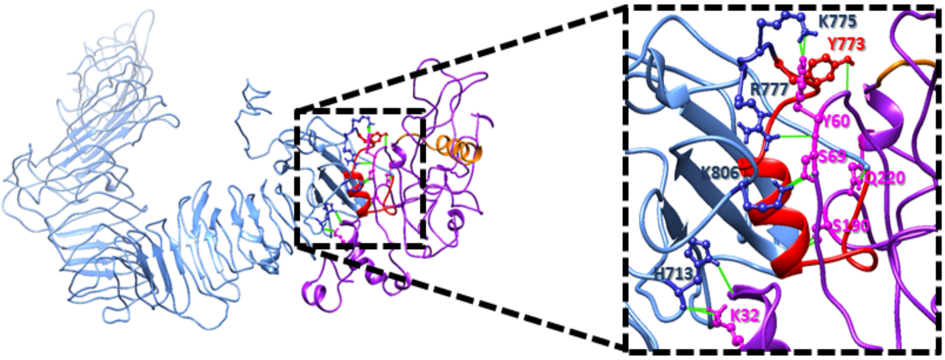

Supplementary Figure 2. Docking of full-length Emerin (light blue) and β-DG (purple) protein structures where their transmembrane domains (Emerin-TM in red, β-DG-TM in orange) are closer to each. Contacting/clashing residues are displayed in *ball-and-stick* conformation (Emerin in magenta, β-DG in dark blue) and their position is also indicated. The interactions (clashes/contacts) are indicated by green lines.
